# Unexpected acute appendicitis found at laparoscopic surgery for a right ovarian teratoma: A case report

**DOI:** 10.1016/j.crwh.2025.e00691

**Published:** 2025-01-31

**Authors:** Rie Okuya, Hiroshi Ishikawa, Nozomi Sakai, Eri Katayama, Kaori Kuroda, Kaori Koga

**Affiliations:** a Department of Obstetrics and Gynecology, Reproductive Medicine, Graduate School of Medicine, Chiba University, Chiba, Japan; b Department of Obstetrics and Gynecology, National Hospital Organization Chiba Medical Center, Chiba, Japan

**Keywords:** Adnexal torsion, Ovarian teratoma, Acute abdomen, Appendicitis, Young adult, Diagnostic imaging

## Abstract

Acute appendicitis and adnexal torsion associated with ovarian tumors are possible causes of acute abdomen in women, yet differentiation remains challenging. Once adnexal torsion is suspected in young women, gynecologists should perform surgery to release the torsion as promptly as possible to preserve future fertility. Herein, we report a case of acute appendicitis that was unexpectedly discovered during laparoscopic surgery initially performed for suspected torsion of a right ovarian teratoma. A 20-year-old nulligravid woman with a history of left ovarian teratoma resection and ulcerative colitis presented with left lower abdominal pain. Non-contrast-enhanced computed tomography showed no findings suggestive of exacerbation of ulcerative colitis, such as bowel wall thickening or worsening ascites, while a mass suspected to be an ovarian teratoma was identified. Gynecologists and an emergency physician specializing in gastroenterology who attended the patient suspected an acute abdomen caused by adnexal torsion or tumor leakage associated with a right ovarian teratoma 5 cm across. However, laparoscopy revealed that the right ovarian teratoma was neither twisted nor ruptured. Instead, the appendix was swollen with yellow turbid ascites, compatible with acute appendicitis. Therefore, laparoscopic excision of the right ovarian teratoma and appendix was performed, and the patient's pain resolved postoperatively. The assumption that the pain was caused by torsion of the right ovarian teratoma was the primary reason for failing to diagnose acute appendicitis. This underscores the importance of evaluating the appendix during gynecologic laparoscopic surgery performed for suspected ovarian torsion.

## Highlights

• Acute appendicitis and adnexal torsion are causes of acute abdomen in women, with differential diagnosis being a challenge.
• Gynecologists tend to consider adnexal torsion as the primary cause of acute abdominal pain.
• Acute appendicitis was identified during laparoscopic surgery for suspected right ovarian teratoma torsion.

## 1 Introduction

Adnexal torsion, mostly associated with ovarian tumors, is a major cause of acute abdomen in women, accounting for 3–5 % of gynecologic emergencies. Gynecologists tend to consider adnexal torsion as the primary cause of lower abdominal pain in women with ovarian tumors. Common ovarian pathologies related to adnexal torsion in young females are functional ovarian cysts and teratoma [1]. Once adnexal torsion is suspected, early surgical intervention to release the torsion and preserve ovarian function should be considered [2]. Ultrasound findings, including ovarian edema, adnexal masses, ovarian Doppler flow, whirlpool sign, and ascites, are useful for the diagnosis of adnexal torsion. However, these findings are not present in all cases, and adnexal torsion is not found during laparoscopy in 50 % of cases [3]. Rather, the identification of an ovarian tumor on imaging in a woman complaining of lower abdominal pain raises the suspicion of adnexal torsion.

The most common non-gynecological cause of lower abdominal pain in females is acute appendicitis [4]; therefore, differentiating between adnexal torsion and acute appendicitis as a cause of acute abdomen is crucial. However, it remains challenging to do so, especially when young women with ovarian cystic tumors present with lower abdominal pain. Despite the presence of an enlarged appendix on computed tomography (CT), physicians may overlook the appendix when an ovarian tumor has already been detected. This is more likely if the patient has a history of contralateral ovarian tumor removal.

The diagnosis and management of a nulliparous woman with suspected right ovarian tumor torsion, which was found to be acute appendicitis, are described.

## 2 Case Presentation

**Fig. 1:**
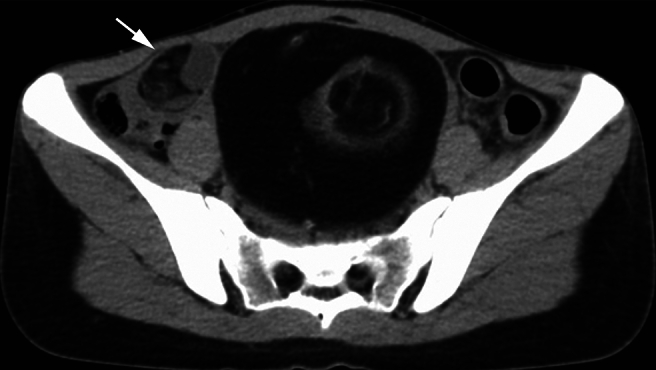
A non-contrast-enhanced computed tomography (CT) scan one year prior to laparoscopic surgery. An ovarian teratoma 10 cm long located in the middle of the pelvic cavity. The tumor contains a significant amount of fatty tissue and a hairball-like structure within the tumor. Another teratoma, 3 cm long, probably arising from the right ovary, is also observed in the same slice (arrow); however, the gynecologists who performed the former ovarian teratoma resection were not aware of the presence of the small teratoma arising from the contralateral ovary.

A 20-year-old nulligravid woman presented at the emergency room with left lower abdominal pain, vomiting, and loose stools. The patient had experienced lower abdominal pain one year previously and underwent a mini-laparotomy to remove a left ovarian teratoma 10 cm long. Upon retrospective examination of a CT scan of that past year, a right ovarian teratoma was noted, 3 cm long (Fig. 1). The follow-up CT scan two months after the surgery also showed the same lesion. Additionally, the patient had a history of ulcerative colitis for 10 months, which was in remission with infliximab therapy.

**Fig. 2:**
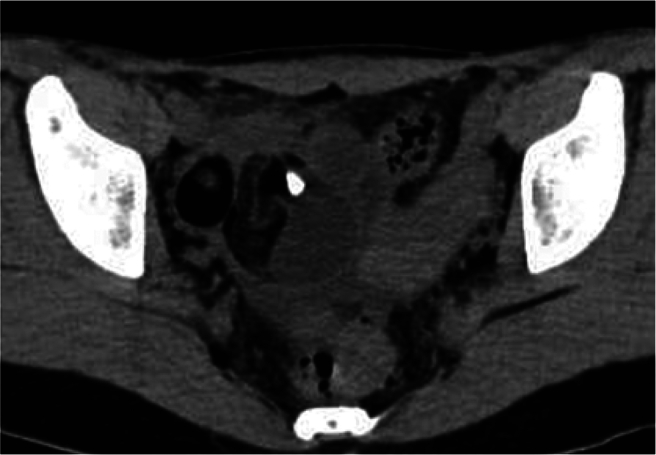
Right ovarian teratoma on CT scan. Preoperative non-contrast-enhanced CT shows a right ovarian teratoma, 5 cm long, containing fat and a calcified part, which had been 3 cm long one year previously, as seen in Fig. 1.

An emergency physician specializing in gastroenterology attended the patient. Her body temperature was 36.2 °C, white blood cell count 14,100/μL (segmented cells 75.9 %), and C-reactive protein level 0.02 mg/dL. Non-contrast-enhanced CT scan revealed a right ovarian teratoma, 5 cm long, and ascites, but no findings suggestive of exacerbation of ulcerative colitis, such as bowel wall thickening or worsening ascites (Fig. 2). Although maximum tenderness was in the right lower abdomen, the patient complained of pain on the left side. Additionally, no peritoneal irritation signs were identified; thus, appendicitis was not strongly considered in the differential diagnosis. Acute exacerbation of ulcerative colitis was also ruled out. Given the presence of a right ovarian teratoma, associated adnexal torsion was mainly suspected. Transvaginal ultrasound performed by a gynecologist revealed a right ovarian teratoma with marked tenderness, and the patient was transferred for emergency surgery.

**Fig. 3:**
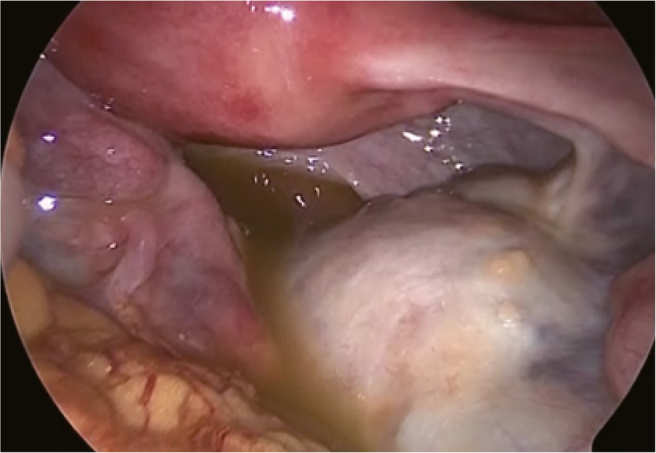
Right ovarian teratoma at laparoscopic surgery. Laparoscopy revealed a right ovarian teratoma with yellow turbid ascites. The right adnexa was neither twisted nor ruptured. (For interpretation of the references to colour in this figure legend, the reader is referred to the web version of this article.)

**Fig. 4:**
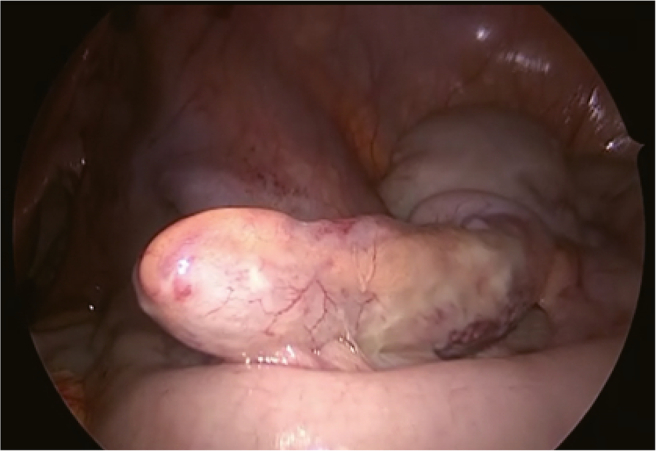
Swollen appendix at laparoscopic surgery. A swollen appendix was detected through routine inspection of the cecum. Subsequently, a gastrointestinal surgeon performed an appendectomy.

**Fig. 5:**
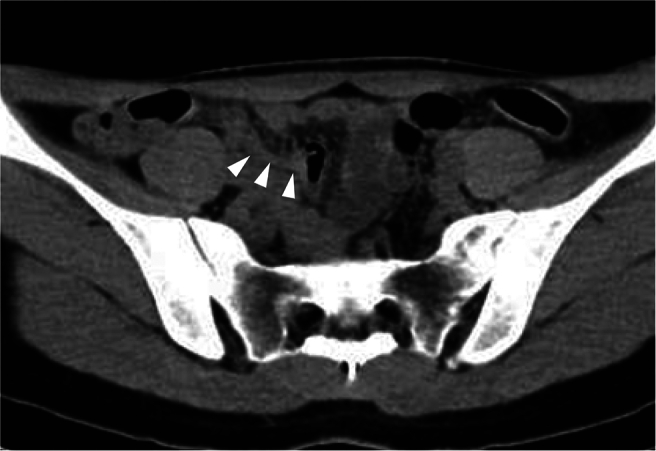
Enlarged appendix on CT scan. Reevaluation of the preoperative CT scan after laparoscopic surgery identifies an enlarged appendix (arrowheads).

Laparoscopy revealed that the right adnexa was enlarged due to the teratoma, but was neither twisted nor ruptured, with yellow turbid ascites (Fig. 3). Furthermore, an enlarged appendix consistent with appendicitis was found (Fig. 4). Based on the laparoscopic findings, retrospective examination of the preoperative CT scan showed an enlarged appendix (Fig. 5). Resection of the right ovarian teratoma and appendectomy were undertaken. Histological examination of the appendix showed marked infiltration of inflammatory cells, with the inflammation extending through all layers of the wall and reaching the serosa. In contrast, the ovarian teratoma showed no histological evidence of neutrophil infiltration or inflammatory spread. Therefore, it was concluded that acute appendicitis was the cause of the acute abdominal pain which had rapidly resolved after the surgery.

## 3 Discussion

In the current case, acute appendicitis, acute exacerbation of ulcerative colitis, and adnexal torsion associated with ovarian teratoma were considered causes of the acute abdomen. The emergency physician primarily considered an acute exacerbation of ulcerative colitis, which was ruled out through the CT scan. Next, a right ovarian teratoma was identified at the point of maximum pain in the right lower abdomen, leading to a suspicion of torsion. Although the swollen appendix was not initially detected on the CT scan, re-examination of the scan confirmed an enlarged appendix.

According to a national survey using an administrative database in Japan, the most common causes of acute abdomen in young females are acute appendicitis and intestinal infections, accounting for 25 % each, while adnexal torsion and rupture of benign ovarian pathologies account for approximately 3–7 % [5]. Similar symptoms of these conditions make differential diagnosis difficult. Although the sensitivity and specificity of CT for diagnosing appendicitis in adults are high, non-contrast-enhanced CT appears to have low sensitivity [6]. In contrast, ultrasound diagnosis of adnexal torsion has much higher sensitivity, and the presence of vascular pedicle twisting and the follicular ring sign are highly associated with a positive ovarian torsion diagnosis, with 100 % specificity [7]. A systematic review evaluating the diagnostic accuracy of ultrasound findings for detecting adnexal torsion revealed that the presence of ovarian edema, whirlpool sign, and decreased or absent ovarian Doppler flow have good specificity and moderate sensitivity for detecting adnexal torsion [3]. A comparison between the test accuracy of ultrasound, CT, and MRI to diagnose adnexal torsion revealed that the pooled sensitivity and specificity of the diagnostic accuracy of ultrasound are 0.79 and 0.76, respectively, and those of MRI are 0.81 and 0.91, respectively [8]. Adoption of an approach that combines ultrasound, CT, and MRI in determining the cause of acute abdomen in young females is needed.

Routine inspection of the abdominal cavity immediately after pneumoperitoneum and before the end of endoscopic procedures, including the surface of the liver, omentum, parietal peritoneum, and cecum, may be useful in gynecological laparoscopic surgery to identify incidental diseases. A cross-sectional study revealed that the rate of incidental findings in abdominal surgery, which included proper inspection of the peritoneal cavity, is estimated to be 1.1 % [9]. In the present case, an enlarged appendix was found during intra-abdominal inspection performed as part of gynecologic laparoscopy and an appendectomy was undertaken. In contrast, the gynecologic surgical team that performed the left ovarian teratoma resection a year previously had not dealt with the right ovarian teratoma at the mini-laparotomy.

In conclusion, the preoperative diagnosis of acute appendicitis in young women with ovarian teratomas remains challenging. Adnexal torsion as a cause of acute-onset abdominal pain should be considered in cases in which an ovarian teratoma is detected.
