# Corrigendum to “Unexpected acute appendicitis found at laparoscopic surgery for a right ovarian teratoma: A case report” [Case Rep Womens Health. 2025 Jan 31:45:e00691]

**DOI:** 10.1016/j.crwh.2025.e00704

**Published:** 2025-03-22

**Authors:** Rie Okuya, Hiroshi Ishikawa, Nozomi Sakai, Eri Katayama, Kaori Kuroda, Kaori Koga

**Affiliations:** a Department of Obstetrics and Gynecology, Reproductive Medicine, Graduate School of Medicine, Chiba University, Chiba, Japan; b Department of Obstetrics and Gynecology, National Hospital Organization Chiba Medical Center, Chiba, Japan

The authors regret an error in the financial support information.

Could you revise the Funding as “This report was partially supported by the KAKENHI 22FB1001.”

The authors would like to apologise for any inconvenience caused.

